# Spore levels in bulk tank organic raw milk and whole milk powder are reduced by udder hair singeing

**DOI:** 10.3168/jdsc.2024-0734

**Published:** 2025-03-18

**Authors:** Zoe D. Wasserlauf-Pepper, Rachel L. Weachock, Christina M. Geary, Martin Wiedmann, Nicole H. Martin

**Affiliations:** 1 Department of Food Science, Cornell University, Ithaca, NY 14853; 2 Quality Milk Production Services, Department of Population Medicine and Diagnostic Sciences, Cornell University, Ithaca, NY 14853

## Abstract

Routine removal of udder hair through singeing or clipping is a farm management practice that is often used with the intention of reducing SCC and mastitis caused by environmental pathogens. Udder hair removal was also recently identified as a factor of importance for the level of bacterial spores in certified organic bulk tank raw milk. Sporeforming bacteria form resistant endospores, or spores, that allow the organisms to survive harsh environmental conditions, including high temperatures, low moisture, exposure to chemicals, and other conditions that would otherwise be lethal to the vegetative cell. Due to this resistance, spores that are present in raw milk can survive processing hurdles used in dairy product manufacturing, germinate into vegetative cells, and ultimately cause spoilage and quality deterioration in dairy products including fluid milk, cheese, and products using dairy powders as ingredients. Therefore, the aim of this study was to determine if udder hair removal through singeing reduces the concentration of bacterial spores in certified organic bulk tank raw milk. Four organic dairy farms that had not routinely removed udder hair were recruited, and bulk tank samples were collected before and after a singeing intervention on all lactating dairy cows contributing to the bulk tank. Raw milk samples, as well as whole milk powder manufactured from the raw milk collected before and after the intervention, were assessed for different groups of dairy relevant bacterial spores. A numerical reduction from before to after intervention was observed in the mean raw milk mesophilic spore count, thermophilic spore count, and butyric acid bacteria most probable number, whereas a numerical increase was observed in the mean raw milk total bacteria count and psychrotolerant spore most probable number, although none of these changes were significant. The mean mesophilic spore count and thermophilic spore count in whole milk powder manufactured from pre- and post-intervention raw milk was, however, significantly reduced from 2.46 to 1.58 log_10_ cfu/g and 1.44 to 1.22 log_10_ cfu/g, respectively. The results of our study indicate that udder hair removal may aid in reducing key populations of spores found in organic raw milk and resulting dairy powders manufactured from that raw milk, although the small sample size in our study likely affected the significance of our results. Future studies should further investigate the role of udder hair removal on a larger set of organic farms, as well as the downstream impact on spore levels in other manufactured dairy products.

## Highlights

• Udder hair removal by singeing is a strategy for improving milk quality.
• A numerical reduction was observed for some groups of spores after hair removal.
• Reductions in mesophilic and thermophilic spores were achieved in whole milk powder.
• Future studies should use a larger sample size to validate the trends seen here.

## Graphical Abstract

**Figure:**
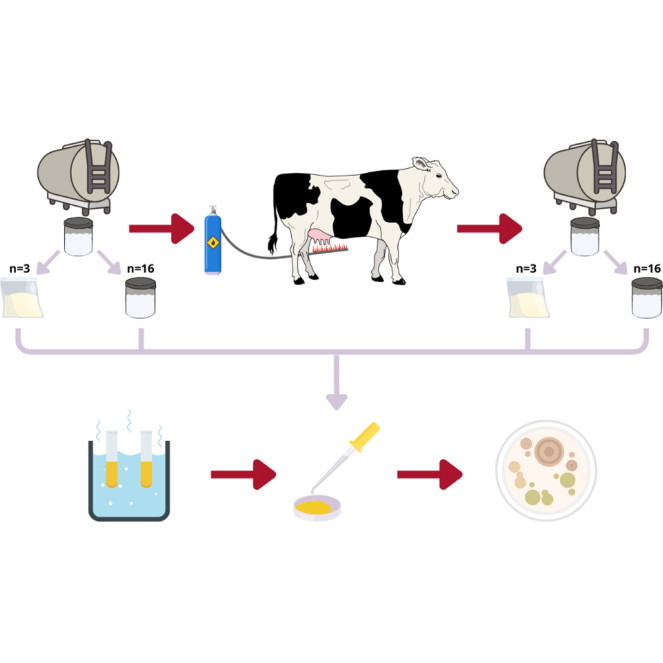
**Summary:** Sporeforming bacteria are important to the dairy industry because they produce resistant endospores that survive conditions that would otherwise be lethal (e.g., pasteurization). Recent research identified udder hair removal as an important factor for the level of spores in organic bulk tank raw milk. To assess the impact of udder hair removal on spores, raw milk was collected before and after udder hair singeing on 4 certified organic dairy farms. Additionally, raw milk from each farm was commingled to manufacture into a batch of pre- and postintervention whole milk powder to evaluate for industry-relevant mesophilic and thermophilic sporeformers. Overall, we observed a trend for reduced spore levels following udder hair removal in bulk tank milk and whole milk powder, although the observed differences were statistically significant in only the whole milk powder. Future studies should increase the number of samples to further elucidate the impact of udder hair singeing on spores.

Organic food product sales continue to increase in the United States, with an estimated $69.7 billion in sales in 2023, with milk and cream sales accounting for a $4.2 billion share (Organic Trade Association, 2024). The quality of dairy products is critical to consumer acceptance, and it is of particular importance for the organic dairy industry to ensure the highest quality products possible, as their distribution channels are often longer than corresponding conventional products due to the reduced processing capacity for organic dairy (Diamond, 2013). Further, organic dairy producers face certain challenges for producing high quality raw milk, namely the restriction on the use of antibiotics, limitations on cleaning and sanitation chemicals allowed for use in the milking system, and increased time of animals on pasture where they become exposed to bacteria present in soil, water, and other natural environments. A key microbial parameter that affects the quality of raw milk is the presence and level of bacterial spores. Sporeforming organisms are unique in that they form resistant spores that withstand harsh environments, including pasteurization, with the possibility of subsequently germinating and growing to concentrations that can cause noticeable quality defects to consumers, thereby limiting the shelf life of processed dairy products. For example, psychrotolerant sporeforming bacteria, predominantly *Paenibacillus* species, that grow at refrigeration temperatures, can reach concentrations in which consumers may detect off-odors or flavors (Huck et al., 2007; Masiello et al., 2014, 2017). Mesophilic and thermophilic sporeformers (e.g., *Anoxybacillus* and *Geobacillus*) are frequently monitored in dry dairy powders (e.g., skim milk powder, whey protein concentrate, and so on) where they may be subjected to stringent specifications, or cause spoilage in products where these powders are used as ingredients (Chen et al., 2004; Watterson et al., 2014; Sadiq et al., 2016). Lastly, anaerobic sporeforming bacteria that produce butyric acid, such as *Clostridium tyrobutyricum,* can cause late blowing in aged cheeses (Klijn et al., 1995; Podrzaj et al., 2022).

Many factors can affect spore levels in bulk tank raw milk, including management practices (e.g., bedding type, frequency of cleaning the bulk tank area) and udder and teat hygiene (Masiello et al., 2017; Martin et al., 2019). Research on bacterial spores in raw milk from organic dairy farms in the United States is limited compared with studies on conventional dairy farms. However, a recent study was conducted on 102 certified organic dairy farms over 1 yr across 11 states, to determine both the prevalence of key dairy-associated spores in organic bulk tank raw milk (**BTM**) and farm factors associated with spore presence and levels (C. Qian, Cornell University, Ithaca, NY; R. T Lee, Cornell University, Ithaca, NY; R. L. Weachock; M. Wiedmann; and N. H. Martin; unpublished data). One farm management practice identified to be important to the level of all spore types evaluated was routinely removing udder hair through clipping or singeing.

Therefore, the goal of this study was to evaluate the effectiveness of udder hair removal to reduce spores in BTM as a means to provide dairy farmers with a practical, rapid, and nonresource intensive method to reduce spores in raw milk. In this preliminary study, we evaluated the concentration of bacterial spores in BTM for 1 wk before and 1 wk after udder hair singeing on 4 organic dairy farms, as well as in whole milk powder (**WMP**) manufactured from the milk collected before and after intervention. This study aims to serve as a proof-of-concept to identify a practical intervention strategy to reduce spores in organic bulk tank raw milk, which should ultimately be reproduced with a larger set of organic dairy farms.

Certified organic dairy farms (A, B, C, and D) located in the northeast United States were recruited from a cohort of 102 certified organic dairy farms that were enrolled in a previous nationwide study concerning bacterial spore levels in organic raw milk (C. Qian, Cornell University, Ithaca, NY; R. T. Lee, Cornell University, Ithaca, NY; R. L. Weachock; M. Wiedmann; and N. H. Martin; unpublished data). Only farms that had not previously routinely removed udder hair were considered for enrollment in the current study. The 4 farms varied by number of lactating cows, milking location, and number of years certified organic (Table 1).

**Table 1:** Characteristics of organic dairy farms (n = 4) enrolled in udder hair singeing intervention study

Farm ID	Number of milking cows	Housing style	Bedding	Milking location	Type of pre- and postdip used	Number of years certified organic
A	41	Stanchions or tiestalls	Straw	Stanchions or tiestalls	Iodine-based	15
B	92	Stanchions or tiestalls	Straw	Stanchions or tiestalls	Iodine-based	26
C	35	Stanchions or tiestalls	Straw	Stanchions or tiestalls	Iodine-based	26
D	96	Bedded pack	Sawdust	Swing parlor	Iodine-based	16

The udder singeing procedure was approved by the Cornell University Institutional Animal Care and Use Committee (#2023-0259), and the experiment was conducted in April 2024. Producers were compensated for participation in this project. All lactating cows whose milk was contributing to the farm's bulk tank during the experimental period were included in the study (Table 1). The singeing procedure was performed as outlined by Strait (2020) with some modification as described here. To perform the udder singeing, a commercial singer (Udder Singe, Armor Animal Health, Beaver Dam, WI) was connected to a portable propane gas source. All eligible cows were flamed immediately after milking either in their tiestalls or in a postparlor holding area. A yellow flame was used on the udder hair, and singeing took between 5 and 15 s, depending on the amount of hair present on the udder. All singeing occurred in a well-ventilated area and both a fire extinguisher and wet towels were kept within reach while the flame was turned on to douse any flare ups. For consistent technique, singeing was performed by one trained technician.

Bulk tank raw milk samples (∼250 mL) were collected by milk truck drivers before every milk pickup for 1 wk before and 1 wk after the singeing intervention on each of the 4 farms, with 4 samples collected from each farm before and 4 samples collected from each farm after the intervention was applied. Samples were immediately frozen at −20°C after collection and then were shipped on ice to the Cornell Milk Quality Improvement Program laboratory (Ithaca, NY). In a previous study, temporary freezing of raw milk samples was shown to have no impact on levels or populations of thermoduric bacteria found in raw milk (Lee et al., 2024). In addition to the 250-mL bulk tank samples, 10 L of BTM was collected from each farm from the milking immediately before and the milking immediately after the intervention (typically within 12 h before and after the intervention). The 10-L pre- and postintervention samples collected from the 4 farms were commingled in a 151-L stainless steel kettle before manufacturing into separate pre- and postintervention WMP for further testing as described below.

The pre- and postintervention commingled BTM was held at <4°C until processing. Pre- and postintervention milk was spray dried in a pilot scale spray drier (Niro, Copenhagen, Denmark) using an inlet temperature of 200°C and outlet temperature of 95°C. The finished product was aseptically transferred into a sterile plastic sampling bag and held at room temperature until testing. The spray drying unit was sanitized between processing pre- and postintervention milk.

Frozen milk samples were thawed in a 6°C incubator overnight before testing. Sample vials were shaken 25 times in 7 s according to Martin et al. (2024) before aliquoting milk for each test. An aerobic plate count (**APC**) was performed by spiral plating 50 μL of raw milk (Neu-tec Eddy Jet 2, Farmingdale, NY) onto standard methods agar (**SMA**) followed by incubation at 32°C for 48 h and then colonies were enumerated using a SphereFlash automated plate counter (Neu-tec, Farmingdale, NY). A 100-mL portion of raw milk was spore pasteurized (**SP**) at 80°C for 12 min in a shaking water bath and then immediately placed in an ice bath (Boor and Martin, 2024). Once the SP milk was cooled to 6°C or below, samples were pour plated by pipetting 1 mL of SP milk into 20 empty petri dish plates and then pouring 12 to 15 mL molten, tempered SMA into each plate and gently mixing. Once agar was solidified, 10 plates were incubated at 32°C for 48 h (mesophilic spore count; **MSC**) and 10 plates at 55°C for 48 h (thermophilic spore count; **TSC**). Colonies were enumerated using an automated plate count reader (Q-count, Advanced Instruments, Norwood, MA). The level of anaerobic butyric acid-bacteria (**BAB**) was estimated using a 20-tube most probable number (**MPN**) method as previously described (Shi et al., 2023). Briefly, 5 mL of raw milk was inoculated into 10 glass tubes filled with 9 mL of Bryant and Burkey broth (**BB**) and 500 μL of raw milk was similarly inoculated into 10 tubes containing 9.5 mL of BB. All tubes were capped with 2-cm paraffin wax plugs and heat treated at 75°C for 15 min. Tubes were stored at 35°C for 6 d at which point they were evaluated for presence of gas production, which was determined by the displacement of the wax plug. All tubes showing no displacement of the wax plug were scored as negative for gas production. The concentration of psychrotolerant spores was also estimated using a 15-tube MPN method, where 5 tubes with 10 mL of SP milk, 5 tubes with 1 mL of SP milk in 9 mL of skim milk broth (**SMB**), and 5 tubes with 100 μL of SP milk in 9.9 mL of SMB were incubated for 21 d at 6°C (Masiello et al., 2014). After incubation, tubes were vortexed, and a 10-μL aliquot of each tube was struck out onto SMA, incubated at 32°C for 48 h, and then checked for bacterial growth. Plates exhibiting growth were scored as positive and plates with no bacterial growth were scored as negative. An MPN table was subsequently used to determine final MPN/L concentration.

To determine spore concentrations in the WMP manufactured from pre- and postintervention milk, 33-g powder samples were taken in triplicate from distinct sections of each of the 2 powder samples using a sterile scoop and placed in 627-mL Whirl-Pak filter bags (Nasco, Fort Atkinson, WI) with 297 mL of PBS. Whirl-Pak bags were stomached for 1 min at 260 rpm. A 25-mL portion was transferred into a sterile glass screw-capped tube and was heated at 80°C for 12 min in a shaking water bath and then immediately placed on ice. Spore-pasteurized reconstituted powder samples were pour plated and incubated for MSC and TSC as described above.

Data were log_10_ transformed before statistical analysis. All data and code used can be found in a publicly available data repository (https://github.com/fsl-mqip/orei-interventions-2024). The Wilcoxon signed rank exact test was used to compare the bacterial spore concentrations for each raw milk test before and after udder singeing paired by farms. The WMP results from this study could not be paired by farm because the raw milk from each farm was commingled before manufacturing, and therefore the Wilcoxon rank sum exact test was used to compare the pre- and postintervention product. All statistical analyses were performed using the stats package in R version 4.3.1 (R Core Team, 2023).

Overall, we observed a numerical reduction from 0.85 to 0.60 log_10_ cfu/mL for MSC, 0.59 to 0.53 log_10_ cfu/mL for TSC, and 2.25 to 2.18 log_10_ MPN/L for BAB from pre- to postintervention; however, these results were not statistically significant (*P* = 0.125, 0.438, and 0.438, respectively; Table 2). Conversely, we observed a slight numerical increase from 3.74 to 3.80 log_10_ cfu/mL in the APC and 1.22 to 1.30 log_10_ MPN/L in the PSC MPN (*P* = 0.438 and 0.563, respectively; Table 2). We observed trends within the data from each of the 4 farms in the study (A, B, C, D), that provide further context to our data. For example, in milk samples collected from farms A, B, and C, we observed a decrease in mean log_10_ APC (ranging from 0.08 to 0.27 log_10_ cfu/mL) and an increase of 0.68 log_10_ cfu/mL in farm D from pre- to post-intervention. Similarly, mean log_10_ MSC from milk samples collected from farms B, C, and D was reduced (ranging from 0.14 to 0.70 log_10_ cfu/mL), whereas an increase was observed in milk from farm A by 0.02 log_10_ cfu/mL from pre- to postintervention. We observed a decrease in mean log_10_ TSC in milk collected from farms A and B (ranging from 0.10 to 0.34 log_10_ cfu/mL) and an increase in mean TSC from milk collected from farms C and D (ranging from 0.01 to 0.21 log_10_ cfu/mL). Mean BAB-MPN log_10_ MPN/L from pre- to postintervention was reduced in milk from farms B and D (ranging from 0.02 to 0.51 log_10_ MPN/L) and increased in milk from farms A and C (ranging from 0.02 to 0.22 log_10_ MPN/L), whereas mean PSC-MPN log_10_ MPN/L was reduced in milk from pre- to postintervention from farms A and D (ranging from 0.07 to 0.16 log_10_ MPN/L) and increased in milk collected from farms B and C (ranging from 0.04 to 0.53 log_10_ MPN/L).

**Table 2:** Mean log concentration of microbial parameters in organic bulk tank raw milk before and after udder hair singeing intervention applied on 4 certified organic dairy farms

Item	Mean^1^		*P*-value^2^
	Pre-intervention	Postintervention	
Aerobic plate count (APC)	3.74	3.80	0.438
Mesophilic spore count (MSC)	0.85	0.60	0.125
Thermophilic spore count (TSC)	0.59	0.53	0.438
Butyric acid bacteria - most probable number (BAB-MPN)	2.25	2.18	0.438
Psychrotolerant spore count - most probable number (PSC-MPN)	1.22	1.30	0.563

1 Log cfu/mL for APC, MSC, TSC; log MPN/L for BAB-MPN, PSC-MPN.

2 *P*-values calculated using a paired Wilcoxon signed rank exact test.

Previous research has investigated intervention strategies for reducing spores in conventional bulk tank raw milk. For example, spore reductions observed in our current study are similar to those reported by Evanowski et al. (2020), that showed mean MSC and TSC were reduced from 0.3 to 0.2 log_10_ cfu/mL, and 0.4 to 0.2 log_10_ cfu/mL, respectively in bulk tank raw milk through a combination of on-farm employee training regarding teat-end cleanliness and procedural changes made to the cleaning and drying of laundered towels used during milking preparation on conventional New York dairy farms. Although spore reductions in our study and others are generally quite small, this is likely due to the exceptionally low initial level of spores in BTM. However, our study provides preliminary evidence that utilizing simple strategies such as udder hair removal may result in reduced spore levels in BTM.

In addition to achieving lower spore concentrations in raw milk, udder hair removal has been recommended to minimize the spread of mastitis within dairy herds (Edmondson, 2012; Stefan and Baraitareanu, 2024). Previous studies involving udder hair singeing primarily focus on its predicted association with SCC (Dufour et al., 2011). For example, in a study evaluating farm management associations with bulk tank somatic cell count (**BTSCC**) on 130 conventional dairy farms in Colombia, the combination of udder hair singeing with the use of pre-dip yielded significantly lower BTSCC compared with farms that did not adhere to those practices (Reyes et al., 2017). Practices that offer multiple benefits (i.e., lower bulk tank spore levels, as well as reduced BTSCC) may be more likely to be adopted by dairy producers; however, a recent study by Qian et al. (C. Qian, Cornell University, Ithaca, NY; R. T. Lee, Cornell University, Ithaca, NY; R. L. Weachock; M. Wiedmann; and N. H. Martin; unpublished data) indicated that only 36% of certified organic dairy producers in the United States include udder hair removal in their management practices. Although the practice of removing udder hair during lactation is broadly recommended, the actual implementation of this practice is variable. In a study by Patel et al. (2019) that examined bedding management practices on 189 conventional dairy farms across 17 states, ∼52.4% of the participating farms reported removing udder hair at least once during lactation. These findings show that there are opportunities to reach producers of various management styles with information on improving milk quality through udder hair removal.

In addition to evaluating the effect of udder hair removal on spores in BTM, we also determined MSC and TSC in WMP manufactured from pre- and postintervention milk to determine the impact of the intervention on downstream dairy products. The mean MSC log_10_ cfu/g in WMP manufactured from milk collected before the singeing intervention was 2.46, whereas the mean MSC log_10_ cfu/g manufactured from milk postintervention was 1.58 (*P* = 0.05; Figure 1). Similarly, we observed a decrease in mean TSC log_10_ cfu/g in postintervention WMP to 1.22 from 1.44 in WMP manufactured from pre-intervention milk (*P* = 0.05; Figure 1). The spore counts in this study were consistent across the discrete samples taken from the manufactured product, with SD of 0.04 log_10_ cfu/g for MSC in both pre- and postintervention WMP, and 0.04 and 0.12 for TSC in pre- and postintervention WMP, respectively (Figure 1).

**Figure 1:**
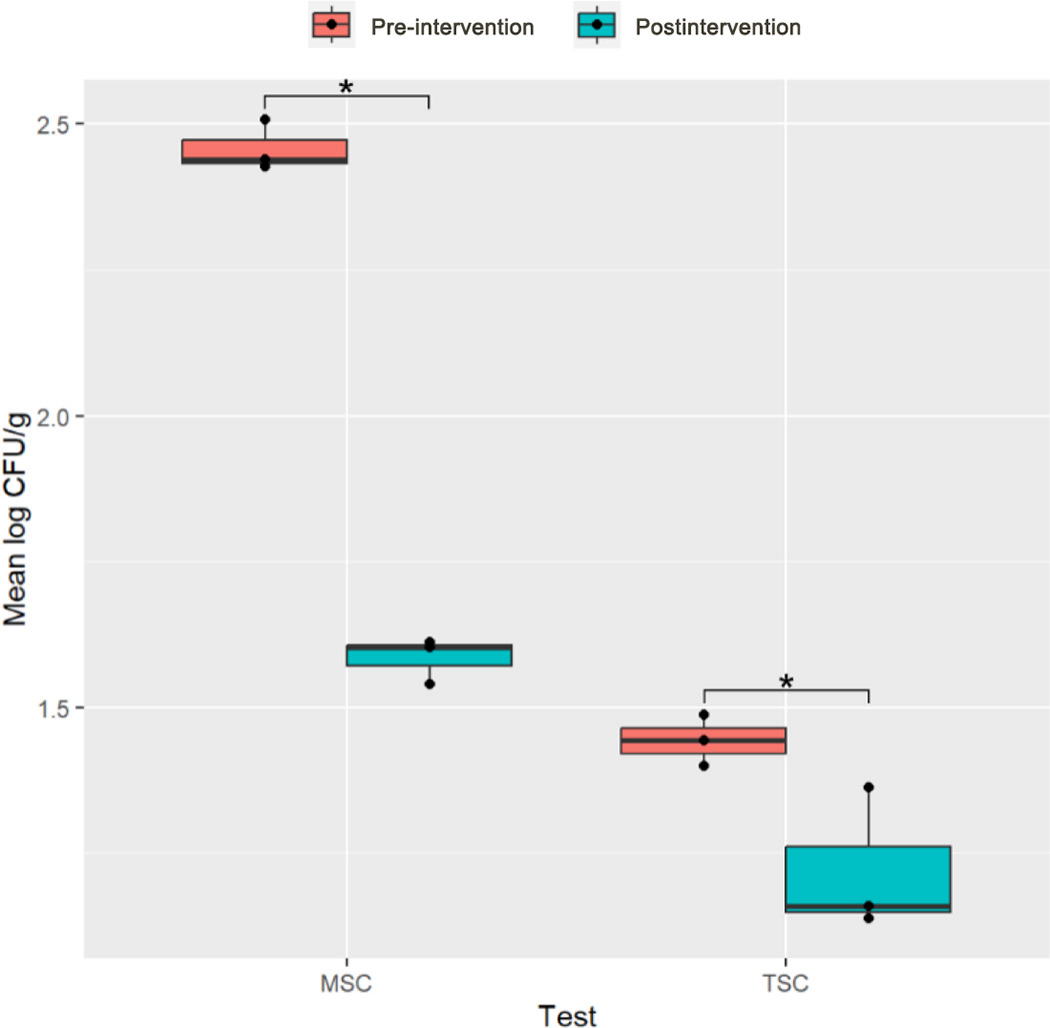
Box plot showing the mesophilic spore count (MSC) and thermophilic spore count (TSC) from 3 distinct 33-g samples within 1 bag of whole milk powder manufactured from pre-intervention (pink) versus 1 bag of postintervention (blue) bulk tank organic raw milk commingled from 4 certified organic producers. The horizontal line within the box represents the median; the lower and upper horizontal edges of the box indicate the first and third quartiles, respectively; and the whiskers extend to 1.5 times the interquartile range. Black dots overlaying the boxes represent individual data points. Asterisks indicate statistical significance using the Wilcoxon rank sum exact test (*P ≤ 0.05).

Mesophilic and thermophilic spores in raw milk contribute to the overall level of spores in dry dairy products as they survive processing conditions and are concentrated in the final product. Spore levels in dry dairy products are often closely monitored, and customers may set stringent specifications, specifically for thermophilic spores (Watterson et al., 2014). For example, limits of as low as 500 cfu/g (2.7 log cfu/g) MSC and TSC have been reported for WMP and skim milk powder (Bienvenue, 2013). Although the manufactured WMP from pre-intervention milk manufactured in our study did not exceed that limit, dairy powder processing facilities with difficulty adhering to their specific tolerance levels may set spore limits for incoming raw milk, encouraging the implementation of management practices linked to lower spore levels in BTM.

Our study provides a preliminary investigation of the effect of udder hair removal on spore levels in BTM, as well as in downstream dairy products manufactured from raw milk. The results of this study are promising as a potential spore reduction intervention; however, this study does have several limitations that should be addressed in future research. Namely, the sample size (n = 4 farms) and the small geographical area used in the study were major limitations and was the likely reason that we did not identify a statistically significant reduction in spores. Further, our data lacked normality, again, likely due to the small number of experimental units, which necessitated the use of nonparametric statistical analyses, which further affected our ability to detect significant changes in spore concentration. Despite these limitations, we believe the outcome of this study demonstrates that udder hair removal by singeing is a rapid, easy, and low-resource practice that should be further studied in relation to the impact on milk quality parameters, specifically bulk tank spore levels and downstream dairy products.
